# EXPANDING HOME-BASED PRIMARY CARE FOR MEDICALLY COMPLEX PATIENTS: RUSH@HOME

**DOI:** 10.1093/geroni/igac059.551

**Published:** 2022-12-20

**Authors:** Walter Rosenberg, Nathaniel Powell, Elizabeth Davis, Leticia Santana, Robyn Golden

**Affiliations:** Rush University Medical Center, Chicago, Illinois, United States; Rush University Medical Center, Chicago, Illinois, United States; Rush University Medical Center, Chicago, Illinois, United States; Rush University Medical Center, Chicago, Illinois, United States; Rush University Medical Center, Chicago, Illinois, United States

## Abstract

Medically complex older adults from underserved communities experience multiple barriers to obtaining necessary health care and services. Often these individuals are largely unable to leave their homes due to a lack of community resources facilitating travel to and from provider appointments. Rush@Home is a home-based primary care program that focuses on the 4Ms by incorporating patient navigation and social work services for medically complex older adults. The majority of the patients enrolled in the Rush@Home program identify as Black and/or Latino (>50%) and reside on the West Side of Chicago which is historically underserved in terms of health care access. To date, Rush@Home has enrolled n=234 participants with n=176 patients who are currently active in the program. Among participants there less than a 10% readmission rate and an ED visit rate of approximately 5%. This presentation will discuss the Rush@Home data and the importance of community engagement.

